# Molecular Epidemiological Analysis of ST11-K64 Extensively Drug-Resistant *Klebsiella pneumoniae* Infections Outbreak in Intensive Care and Neurosurgery Units Based on Whole-Genome Sequencing

**DOI:** 10.3389/fmicb.2021.709356

**Published:** 2021-09-27

**Authors:** Liuxin Xiong, Lebin Su, Hanqing Tan, Wansha Zhao, Shuying Li, Yingmei Zhu, Limiao Lu, Zhiwei Huang, Baisheng Li

**Affiliations:** ^1^Clinical Laboratory, The Second People’s Hospital of Zhaoqing, Zhaoqing, China; ^2^Microbiological Laboratory, Zhaoqing Center for Disease Control and Prevention, Zhaoqing, China; ^3^Institute of Microbiology, Guangdong Provincial Center for Disease Control and Prevention, Guangzhou, China

**Keywords:** extensively drug-resistant *Klebsiella pneumoniae*, whole-genome sequencing, virulence resistance factors, drug resistance factors, nosocomial infection, outbreak

## Abstract

*Klebsiella pneumoniae* (Kp) is the primary causative bacteria for nosocomial infections and hospital outbreaks. In particular, extensively drug-resistant *K. pneumoniae* (XDRKp) causes severe clinical infections in hospitalized patients. Here, we used pulsed-field gel electrophoresis (PFGE), drug susceptibility tests, and the whole-genome sequencing (WGS) technology to examine genetic relatedness and phenotypic traits of the strains isolated during an outbreak period. Based on PFGE, a distinct clones cluster comprised of eight XDRKp was observed. These strains were confirmed as ST11-K64 *via* multiple-locus sequence typing database of Kp. The strains also had genes related to the regulation of biofilm biosynthesis (type 1 & 3 fimbriae, type IV pili biosynthesis, *RcsAB*, and type VI secretion system) and multiple drug resistance (β-lactamase and aminoglycoside antibiotic resistance). WGS data based on core-single nucleotide polymorphisms and epidemiological investigation showed that the neurosurgery unit was likely the source of the outbreak, the strain was likely to have been transmitted to the ICU through patients. In addition, the two highly probable transmission routes were in the ICU (exposure through shared hospital beds) and the neurosurgery units (all cases were treated by the same rehabilitation physician and were most likely infected during the physical therapy). Notably, the bed mattress had played a crucial transmission role of this outbreak, served as a pathogen reservoir.

## Introduction

*Klebsiella pneumoniae* (Kp) is a Gram-negative enteric bacillus belonging to the *Enterobacteriaceae* family and can cause several infectious diseases, such as pneumonia, urinary tract infection, and bloodstream infection. It is the most common pathogen associated with nosocomial infections (Gorrie et al., 2017). However, multidrug resistance *Klebsiella pneumoniae* (MDRKp) and drug-resistant *Klebsiella pneumoniae* (XDRKp) lead that it is significantly difficult to choose appropriate antibiotics to treat its hospital-acquired infections (Davies and Davies, 2010). Other studies have shown that they cause severe complications after infection, bringing significant challenges to existing clinical medication (Giske et al., 2008; Shiri et al., 2017).

In China, several studies have highlighted that ST11 is the dominant clonal group of carbapenem-resistant *K. pneumoniae* (CRKp; Qi et al., 2011; Sui et al., 2018; Bing et al., 2019; Liu et al., 2019; Yu et al., 2019a; Zhou et al., 2020a). Moreover, existing mobile genetic elements in some ST11 CRKp genomes have enhanced their survival adaptability and provided a prerequisite for their global prevalence (Li et al., 2020).

Although traditional molecular typing technology can accurately define and identify the specific outbreak strains, it cannot reveal the transmission relationship between the outbreak strains and the potential source(s). Recently, whole-genome sequencing (WGS) has been widely used in genetic evolution, population migration, and epidemic analysis of pathogenic bacteria (Mutreja et al., 2011; Croucher et al., 2013; Leekitcharoenphon et al., 2014). Subsequently, outbreak investigations and epidemiological analyses have preferably applied WGS to evaluate disease prevention and control measures (Gardy et al., 2011; Van Ingen et al., 2017; Sui et al., 2018; Chen et al., 2019).

Herein, we used WGS data in combination with epidemiological data to analyze the source of the outbreak in the ICU and neurosurgery unit caused by the ST11-K64 XDRKp strains. They were also used to reconstruct the most likely transmission routes of the outbreak for effective prevention and control measures. Besides, the virulence and drug resistance genes of the outbreak strains were determined to explain the poor clinical antibiotic treatment effect and serious infection in the infected patients. This study provides insights into the ST11-K64 XDRKp strain, raising awareness of its infection.

## Materials and Methods

### Collection of Strains and Analysis of Epidemiological Data

An outbreak of Kp occurred in hospital ICU and neurosurgery unit in Zhaoqing City, Guangdong Province, China from April 2, 2021 to June 29, 2021. To study the genetic relatedness and phenotypic traits of the strains isolated during the outbreak period, 33 Kp strains comprised of isolated from February 3, 2021 to July 20, 2021 were included in this study. Patients data including admission time, department, bed, isolation time, and diagnosis were collected.

### Strains Isolation, Identification, and Drug Sensitivity Test

All strains were identified using the VITEK2 compact automatic bacterial identification and drug sensitivity analysis system (BioMérieux, Lyon, France). A wire drawing test (Nadasy et al., 2007) was used to confirm the hypermucoviscosity (HM) phenotype of the strains. Briefly, a fresh colony cultured overnight on the blood agar plate was gently touched with an inoculation ring and pulled outward twice. HM phenotype was positive (HM phenotype positive) if the mucilaginous filaments (5mm long) were formed. Minimal inhibitory concentrations (MIC) of 23 antibiotics were determined (DL Biotech, Zhuhai, China) based on the national pathogen identification network monitoring manual protocol. The results were interpreted according to the 2020 Clinical and Laboratory Standards Association (CLSI-M100-S30) guidelines. Lastly, the quality control strains of biochemical identification and drug sensitivity test of *Escherichia coli* (*ATCC*25922) and *K. pneumoniae* (*ATCC*700603) were performed.

### Pulsed-Field Gel Electrophoresis

Related bacterial strains were inoculated on blood agar plates and cultured overnight at 37°C. After obtaining a pure culture, a loopful of bacteria was taken from the colony and transferred into a Falcon 2054 test tube containing 2ml TE. The turbidity of the suspension was adjusted to 3.0–4.2 before use. Subsequently, 400μl bacteria suspension was digested with 5μl of 10mg/ml protease K solution (Promega, Fitchburg, United States) and incubated at 37°C for 5min to prepare a DNA gel block. Then, the restriction enzyme *Xba I* (Promega, Fitchburg, United States) was used to digest *K. pneumoniae* and *Salmonella reference strain* (H9812) gel blocks, using the electrophoresis CHEF MAPPER (Bio-Rad, Laboratories, United States). The electrophoresis conditions included as: voltage 6.0V/cm, pulse parameters 6–36s, and electrophoresis for 19h. After ethidium bromide staining, GelDocXR+ (Bio-Rad, Laboratories, United States) was used to capture images, and bionumerics version 7.1 was used for all image analysis (Applied Maths, Kortrijk, Belgium; Han et al., 2013).

### DNA Extraction, Sequencing, and Assembly of Strains

Genomic DNA was extracted using QIAamp DNA Mini Kit (Qiagen, Dusseldorf, Germany) according to the manufacturer’s instructions. The purity of genomic DNA was validated by NanoDrop1000 ultra microspectrophotometer (Thermo Fisher, Waltham, United States) and quantified by Qubit DNA-HS (Thermo Fisher, Waltham, United States) assay. The average genomic DNA concentration was 135ng/μl, and the final volume was 100μl. The extracted genome was sequenced on the MiSeq (Illumina, San Diego, United States) platform. Next, the Nextera XT DNA Library Preparation Kit (Illumina, San Diego, United States) was used to construct the gene library. Afterward, the sequenced reads were assembled using the *de novo* mode on the CLC Genomics Workbench version 9.5.3 (Qiagen, Dusseldorf, Germany) platform (Lauraine et al., 2018). The raw sequencing data were qualified by removing 5 BP fuzzy base, bases with quality scores <20 and reads shorter than 20bp, eliminating adapter pollution, and removing repeated reads. The assembled contacts /scaffolds were first analyzed using the Rast online gene annotation analysis tool (Overbeek et al., 2013),^1^ and the virulence genes were identified using VFBD (Liu et al., 2018).^2^ Lastly, the drug resistance genes were identified using CARD (McArthur et al., 2013).^3^

### Nucleotide Sequence Accession Number

This whole-genome shotgun project has been deposited at GenBank under the Bioproject ID PRJNA715018 and the following accessions: JAGFMM000000000, JAGFMN000000000, JAGFMO000000000, JAGFMP000000000, JAGFMQ000000000, JAGFMR000000000, JAGFMS000000000, and JAGFMT000000000.

### Multi-locus Sequence Typing and Whole-Genome Analysis

The WGS data of Kp strains were submitted to the multiple-locus sequence typing (MLST) database.^4^ The sequence type (ST) was determined by identifying seven housekeeping genes (Diancourt et al., 2005). *wzi* and *wzc* genes were identified to determine the capsular type (Brisse et al., 2013; Pan et al., 2013). The HemI 1.0 software was then used to visually analyze the drug resistance spectrum and related genes (Deng et al., 2014). Concurrently, the kSNP version 3.0 software (Gardner and Hall, 2013) was used to construct the core-single nucleotide polymorphisms (core-SNPs) evolutionary tree. The kchooser tool of the kSNP version 3.0 software was first used to calculate the whole genome of eight strains by using the k-mer algorithm and maintaining all other parameters in default. This study calculated the k-mer value was 21, and all the core-SNPs were identified using this value as the calculation parameter. Finally, the core-SNPs were used to compose the sequence. The evolutionary tree was constructed using the maximum likelihood method to reconstruct the transmission chain based on genomic information and case epidemiological data.

## Results

### Analysis of Epidemiological Data

**Figure 1:**
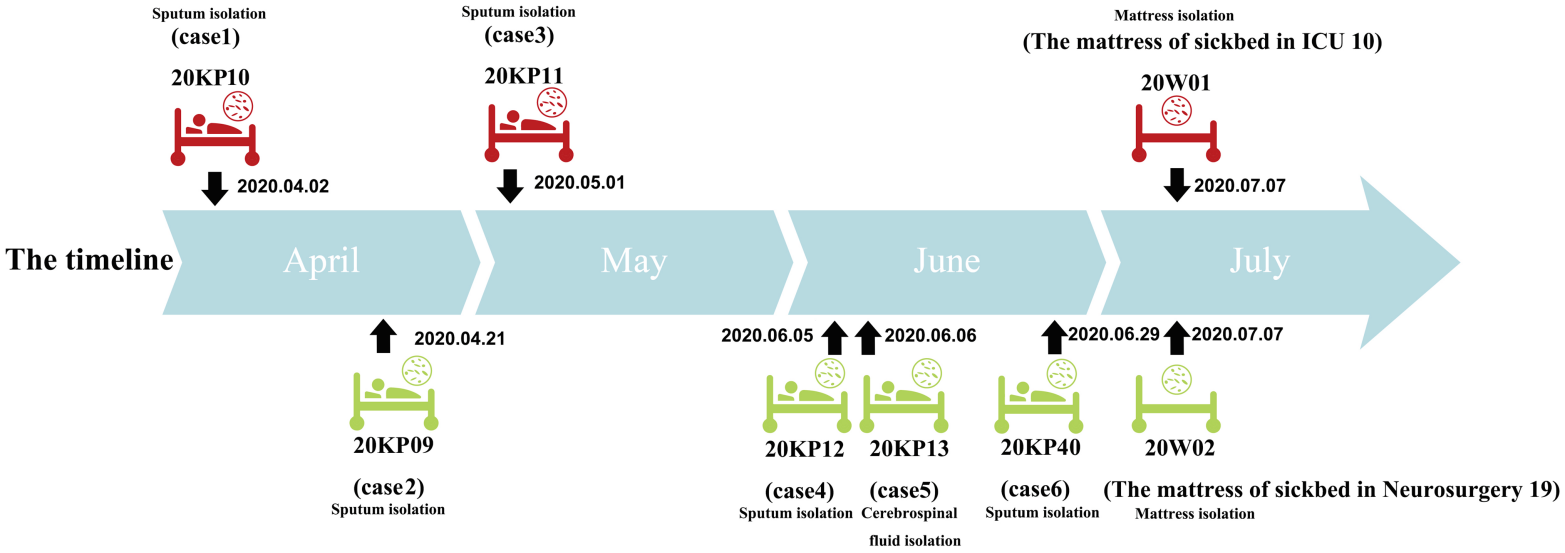
Timeline of isolation of the eight drug-resistant *Klebsiella pneumoniae* (XDRKp) strains in this outbreak. The red pattern represents isolation from the ICU, and the green pattern represents isolation from the neurosurgery unit. The date next to the black arrow are the isolation date of the strain.

Herein, six patients were diagnosed with Kp infection. The outbreak began on April 2, 2020, when the strain (20KP10) was first isolated from the sputum of an ICU patient (case 1). More Kp strains with the same phenotype and morphology were isolated from the body fluids of case 2 to case 6 in the subsequent 4months (Figure 1). One of the six infected patients died (case 1), and the other infected patients were transferred to a superior hospital for further treatment due to the unsatisfactory treatment effect. Five infected patients were over 40years old. Epidemiological investigation showed that all the infected patients had basic diseases, surgical history, tracheal mechanical incision history, and severe pneumonia after infection. All the cases were correlated, i.e., used one hospital bed (case 1, case 2, case 3, and cases 6), were in adjacent beds (case 2 and case 6), or were treated with the same physical therapist (case 2, case 4, case 5, and case 6; Table 1). Therefore, the hospital’s nosocomial infection prevention and control department immediately conducted two sampling tests on the outside environment of the infected patients and the hands of medical staff. The first external environment test did not detect Kp. However, the second test detected Kp on the samples of the mattresses on the bottom of two beds. The drug susceptibility test showed that the drug-resistant phenotype was XDRKp. Notably, the colony phenotypes of the eight strains isolated were all non-mucoid (shorter than 5mm, and the wire drawing test was negative).

**Table 1:** Epidemiological information of the six infected patients.

Case	Admission Time	Bed number and Residence time of the patient		Age	Clinical Basic diseases	Postinfectious disease	Department	Rehabilitation physical therapy	Whether administered by the same rehabilitation physician	Discharge status
Case 1	2020.01.04	Respiratory unit Sickbed 32	2020.01.04-2020.03.19	86	Hypertension, Multiple pulmonary bullae in both lungs	Severe pneumonia, Type II respiratory failure	Respiratory unit, ICU	No	No	Died
		ICU Sickbed 10	2020.03.20-2020.04.10							
		ICU Sickbed 08	2020.04.11-2020.06.01							
Case 2	2020.03.15	Emergency unit Sickbed 20	2020.03.15	53	Sequelae of cerebral infarction, Type II diabetes mellitus	Severe pneumonia	ICU, Neurosurgery unit	Yes	Yes	Transferred to a more advanced hospital
		ICU Sickbed 08	2020.03.16-2020.04.01							
		Neurosurgery unit Sickbed 18	2020.04.01-2020.05.16							
Case 3	2020.04.04	Digestive Medical Ward Sickbed13	2020.04.04-2020.04.10	92	Left thalamus hemorrhage, Hypertension	Severe pneumonia, Intracranial infection	ICU Digestive Medical Ward	No	No	Transferred to a more advanced hospital
		ICU Sickbed 10	2020.04.11-2020.04.17							
		Digestive Medical Ward Sickbed 1	2020.04.18-2020.04.29							
		ICU Sickbed 10	2020.04.30-2020.05.06							
Case 4	2020.05.04	Neurosurgery unit Sickbed 3	2020.05.04-2020.06.11	43	Brain aneurysm, Refractory epilepsy	Severe pneumonia	Neurosurgery unit	Yes	Yes	Transferred to a more advanced hospital
Case 5	2020.05.04	ICU Sickbed 06	2020.05.04-2020.05.24	13	Cerebral vascular malformation, Secondary epilepsy, Spastic quadriplegia	Severe pneumonia, Brain abscess	ICU, Neurosurgery unit	Yes	Yes	Transferred to a more advanced hospital
		Neurosurgery unit Sickbed 37	2020.05.25-2020.07.16							
Case 6	2020.05.09	ICU Sickbed 10	2019.07.24-2019.08.17	71	Sequelae of cerebral infarction, Hypertension	Severe pneumonia, Acute cholangitis	ICU, Neurosurgery unit	Yes	Yes	Transferred to a more advanced hospital
		Neurosurgery unit Sickbed 19	2020.05.09-2020.07.01							

### PFGE, MLST, and Capsule Typing

**Figure 2:**
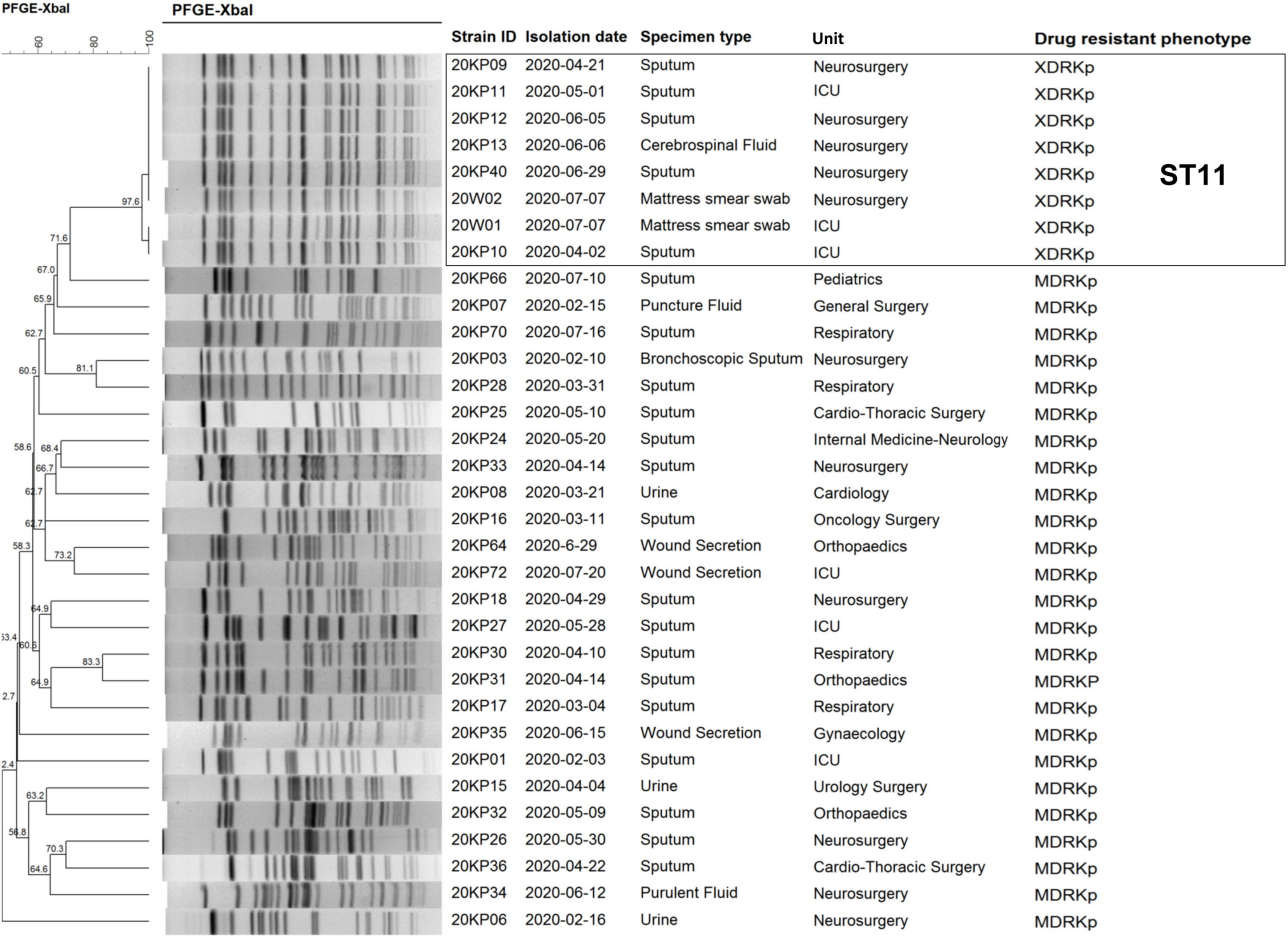
Clustering map of 33 *Klebsiella pneumoniae* strains by pulsed-field gel electrophoresis.

Pulsed-field gel electrophoresis typing was performed on eight XDRKp strains and 25 MDRKp strains isolated from the hospital before and after the outbreak to screen the different cases related to the outbreak. Our findings (Figure 2) revealed eight XDRKp strains in this outbreak, all of the same clone. In addition, at least 10 bands that were different from those of XDRKp were also isolated in this hospital. However, there was no apparent genetic relationship between the outbreak and clinical isolates. After comparing the whole genome using the MLST database of Kp, the isolated strains were determined as ST11, whereas the capsule type was K64.

### Identification of Virulence Factors

The various virulence genes factors of the whole genome of XDRKp strains were identified using VFBD. Here, all the eight XDRKp strains had 80 virulence genes (Supplementary Material 1), of which all genes had 79. Besides, all these strains carried genomes related to biogenesis (type 1&3 fimbriae, type IV pili biosynthesis, *RcsAB*, type VI secretion system (T6SS), etc.). According to the annotation on VFBD, thes protein products encoded by the virulence genes belonged to seven different functional groups (adherence, antiphagocytosis, efflux pump, iron uptake, regulation, secretion system, and serum resistance group). Furthermore, the *pilu* gene encoding the twitch motor protein PilU and owned by *Pseudomonas aeruginosa* was discovered in the genomes of the eight XDRKp strains. Notably, *rmpA* and *rmpA2* genes involved in the regulation of mucus phenotype, and *colistin* genes were not detected in the genome of the eight strains.

### Drug Sensitivity Test and Drug Resistance Gene Identification

**Figure 3:**
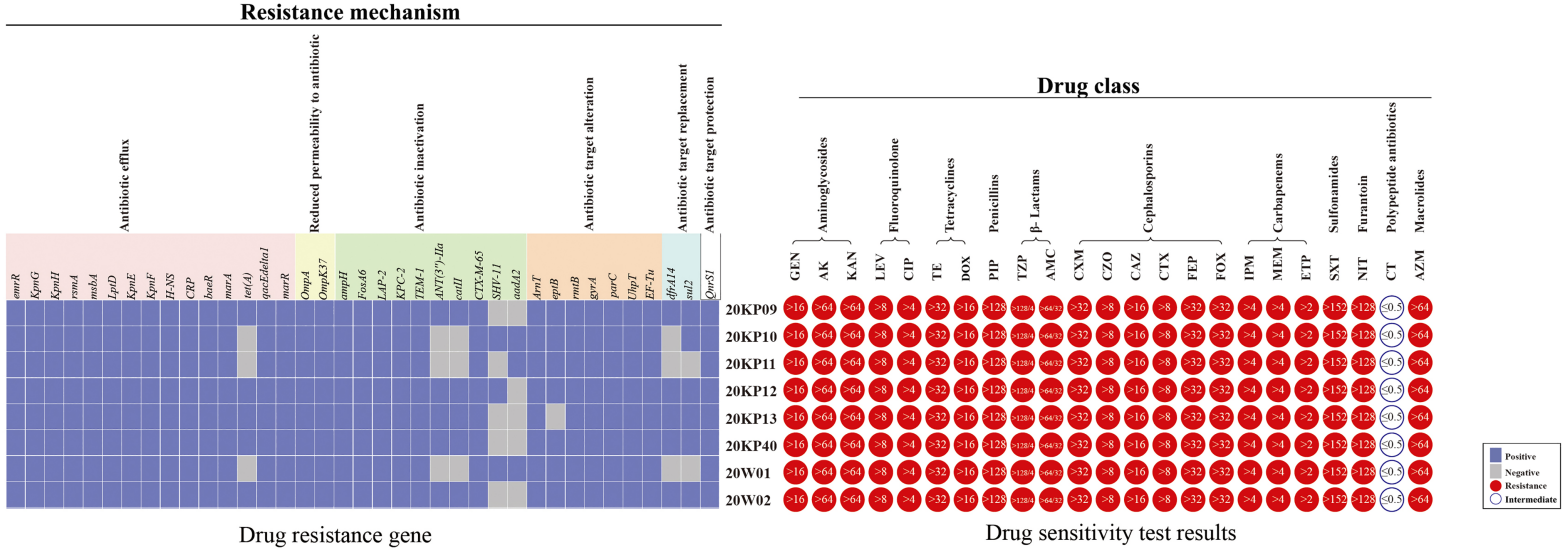
The drug resistance gene distributions and antibiotic resistance spectrum of eight XDRKp strain. The 23 drugs in the drug sensitivity test included Gentamicin (GEN), Amikacin (AK), Kanamycin (KAN), Levofloxacin (LEV), Ciprofloxacin (CIP), Tetracycline (TE), Doxycycline (DOX), Piperacillin (PIP), Piperacillin/Tazobactam (TZP), and Amoxicillin/Clavulanicacid (AMC). Cefuroxime (CXM), Cefazolin (CZO), Ceftazidime (CAZ), Cefotaxime (CTX), Cefepime (FEP), Cefoxitin (FOX), Imipenem (IMP), Meropenem (MEM), Ertapenem (ETP), Trimethoprim/sulfamethoxazole (SXT), Nitrofurantoin (NIT), Colistin (CT), and Azithromycin (AZM). The value in the circle of the drug sensitivity test results is the MIC value of the strain.

Antibiotic susceptibility tests (23) on the numerous strains of this outbreak showed that the eight strains had similar drug sensitivity outcomes. The strains were resistant to 22 antibiotics, including aminoglycosides, quinolones, tetracyclines, β-lactams, cephalosporins, hydromolems, sulfonamides, furantoin, and intracyclic lipids. The MIC values were all higher than the drug concentrations recommended by CLSI-M100-S30 for the antibiotic sensitivity test. However, the sensitivity to colistin had a MIC value ≤0.5 (Figure 2). This type of strain was determined to be XDRKp through bioantibiotic susceptibility test. The genomes of the eight strains were uploaded to the CARD database, and 37 drug-resistant genes were identified. The information of their gene sequences and drug resistance mechanism was found in the CARD database (Supplementary Material 2), consistent with the drug resistance phenotypes of nine classes of antibiotics detected in the antibiotic sensitivity test. Notably, *OqxAB*, *nfsA*, and *nfsB*, which regulate nitrofurantoin resistance, were not detected, but the strains had a resistance phenotype to nitrofurantoin. All the strains had 29 of the 37 resistant genes, including five β-lactamase genes (*BLA_ampH_*, *BLA_LAP-2_*, *BLA_KPC-2_*, *BLA_TEM-1_*, and *BLA_CTX-M-65_*) and aminoglycoside resistance genes (five genes encoding efflux pump (*KpnG*, *KpnH*, *KpnE*, *KpnF*, and *baeR*) and one gene encoding antibiotic target change (*rmtB*)). *eptB* gene was detected in all strains except for 20KP13. The other seven drug-resistant genes (*sul2*, *tet(A*), *ANT (3')-IIa*, *catII*, *dfrA14*, *SHV-11*, and *aadA2*) were different among the eight XDRKp strains, mainly due to the variations among 20KP10, 20KP11, 20W01, and the other 5 strains (Figure 3).

### Evolution Analysis and Propagation Chain Inference of Outbreak Isolates Based on Core-SNPs

**Figure 4:**
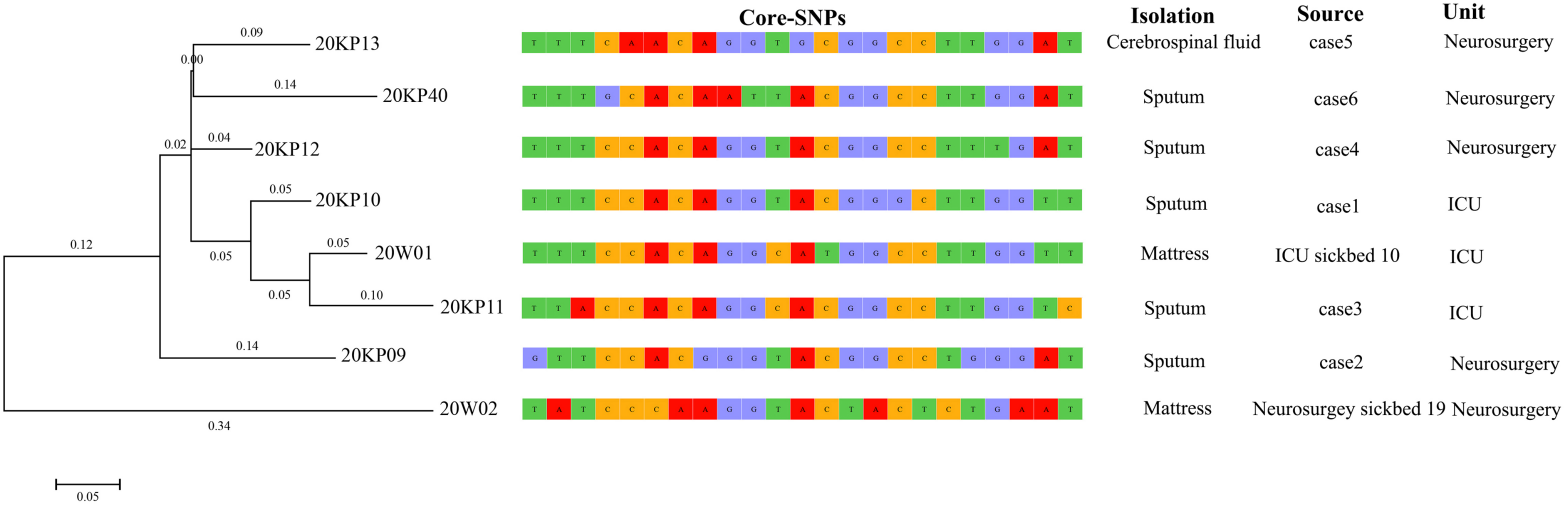
Phylogenetic tree of eight XDRKp strains based on core single-nucleotide polymorphisms.

**Figure 5:**
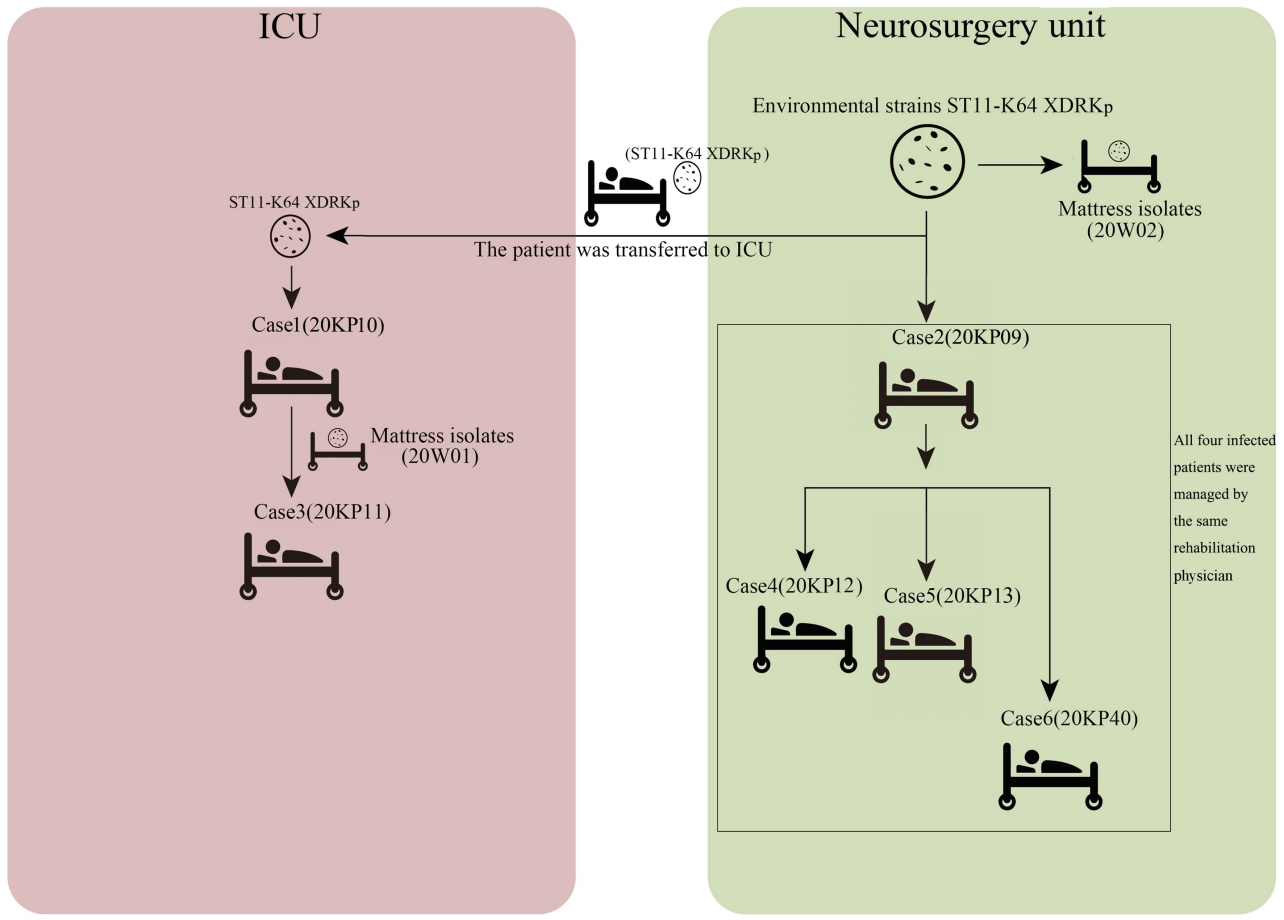
Transmission chain of eight XDRKp strains.

The WGS analysis revealed the presence of only 23 core-SNPs in the eight XDRKp strains. The 20KP11 vs. 20W02 strains had the highest difference (12) in core-SNPs. However, some strains, such as 20KP10 vs. 20KP12, 20KP10 vs. 20W01, and 20K11 vs. 20W01, had a difference of only three core-SNPs. Therefore, the eight strains are closely related. On the other hand, phylogenetic analysis of core-SNPs based on the k-mer algorithm showed that the eight strains were divided into two evolutionary branches (Figure 4). Branch 1 only had the 20W02, and branch 2 had the other seven strains. The seven strains in branch 2 produced four small evolutionary clades, including clade A (20KP09), clade B (20KP13 and 20KP40), clade C (20KP12), and clade D (20KP10, 20KP11, and 20W01). By combining genomic evolution information and case epidemiological information, this study inferred two transmission chains: one in the ICU and the other in the neurosurgery unit. In the ICU, case 1 transmitted this type of strain to case 3 through the contaminated hospital bed, whereas in the neurosurgery unit, case 2 transmitted the strain to cases 4, 5, and 6 during physiotherapy (Figure 5).

## Discussion

Herein, nosocomial infections of XDRKp identified in the ICU and neurosurgery units causing one death and five severe cases. All six patients had a history of surgery and mechanical tracheal incision, and the severe pneumonia was observed following the infection (Table 1). Some patients also had Type II respiratory failure (case 1), intracranial infection (case 3), brain cyst (case 5), and multiple organ dysfunction syndrome (case 6). These observations indicate that the strain mainly infected middle-aged and elderly patients in the hospital, causing severe clinical symptoms and even death. The strain was detected in ICU and neurosurgery units indicating the contamination in the external environment of the two clinical units.

According to the PFGE interpretation standard model proposed by Tenover et al. (1995) all the eight strains were “the same” or “closely related” (Tenover et al., 1995; Figure 1). All the strains were identified as the same clones. However, they were not related to the other 25 MDRKp strains isolated in the hospital. Besides, PFGE could not construct the transmission route of the epidemic and trace the source of the epidemic caused by the eight strains. However, PFGE was used to compare electrophoretic band clustering between the patient isolates and the environmental isolates. The results suggested that the environmental strains in the bed mattress could be the source of the infections in ICU. Based on the MLST analysis, our findings showed that all the strains were ST11, and the capsule type was K64. Notably, the ST11 strain has caused many hospital infections in China (Zhan et al., 2017; Gu et al., 2018; Chen et al., 2019; Yu et al., 2019b), and it is also the CRKp dominant clonal group in China. The capsule K64 type is less reported than the common mainstream K1 and K2 types (Zhang et al., 2016). Different strains with the ST11-K64 combination have been recently reported and could develop into new highly resistant super bacteria (Zhang et al., 2016; De Campos et al., 2018; Yang et al., 2020).

Herein, all the strains isolated were non-mucoid. The virulence of the eight strains was weaker than the previously reported ST11-K64 strains due to the lack of mucus phenotypic regulatory *rmpA*, *rmpA2*, and *colistin* genes (Zhang et al., 2016; De Campos et al., 2018; Yang et al., 2020; Zhou et al., 2020b). Notably, this type of strain had various regulatory and biofilm-related genomes, such as type 1&3 fimbriae, type IV pili biosynthesis, *rcsAB*, and T6SS (Supplementary Material 1). Therefore, the eight strains can synthesize strong biofilms, thus greatly increasing the adaptability of the strains to the environment or host. In particular, the *rcsAB* and T6SS genes have been reported to greatly enhance the cell invasion and *in vivo* colonization ability of the strain (Peng et al., 2018; Barbosa and Lery, 2019). The eight strains were resistant or intermediary to the antimicrobial drugs recommended based on CLSI-M100-S30. Notably, these strains had a phenotype of resistance to furantoin. The *OqxAB*, *nfsA*, and *nfsB* genes regulating the resistance of furantoin were not detected in the strains (Sandegren et al., 2008; Ho et al., 2016). However, further studies are needed to determine whether the strains have other mechanisms for regulating furantoin resistance. Besides, efflux pump, cellular pore protein, antibiotic inactivation, antibiotic target change, targeted replacement, and targeted protection genes were detected in the eight strains. These strains all had five β-lactamase genes. This study also suggested that the strong drug resistance led it was difficult for the clinical anti-infection treatment and resulted poor prognosis in patients. The subsequent treatment effect of the six patients also showed similar results. Also, the eight drug-resistant genes (*sul2*, *tet(A*), *ANT(3'')-IIa*, *catII*, *dfrA14*, *SHV-11*, *eptB*, and *aadA2*) were mainly detected in the eight strains were different. Further studies are needed to determine whether the variance in drug resistance genes was due to the difference in the antibiotic environment during the transmission.

Herein, 20KP10 was first isolated from the sputum of an ICU patient (case 1). seven strains with the same phenotype and morphology were isolated from the body fluids and the environment in the following 4months (Figure 1). The analyses on the epidemiological data and the genomic data showed that there were two transmission chains (Figure 5) found in the ICU and neurosurgery units. In the ICU, case 1 was likely to transmit the strain to case 3 through the contaminated hospital bed. In the neurosurgery unit, case 2 may transmit the strain to cases 4, 5, and 6 during physiotherapy. The source of the isolates was also assessed. First, although the strain was first detected in ICU, it was not the source of the outbreak. Case 1 was the first case of infection and only transmitted the strain to case 3. The two cases (1 and 3) had indirect contact (ICU sickbed 10), and there were only three core-SNPs in the three isolates (20KP10, 20KP11, and 20W01). The D branch was at the end of the evolutionary tree, indicating that the three isolates from ICU evolved later after other isolates. Therefore, the ICU epidemic could be due to vertical transmission. Second, the epidemiological data (Table 1) showed that case 6 had been admitted to ICU bed 10 7months before the outbreak. At that time, the bed was not infected with this strain, and case 6 patient was only infected when he was admitted to the neurosurgery unit in 2020. Moreover, this strain had not been isolated from other patients in the ICU before the infection in case 1, indicating that the strain was not in the ICU before this outbreak. Third, although the 20W02 strain was isolated on July 7, 2020, in neurosurgery 19 mattress, it was located in the root of the tree. It had at least 11 base differences with the other seven strains in core-SNPs. It had the longest evolutionary branch distance, indicating that the strain was in the external environment of the neurosurgery unit before the outbreak and took longer to evolve. Therefore, the first occurrence of this strain could have been in the neurosurgery unit. The origin of this strain in the ICU could have happened during a case transfer between the neurosurgery unit and the ICU. Also, the outbreak in the neurosurgery unit could have originated from case 2 since the evolutionary node of 20KP09 occurred earlier than that of the other three isolates (20KP12, 20KP13, and 20KP40). Moreover, all four cases were handled by one physician, and rehabilitation physical therapy was first conducted in case 2. Therefore, case 2 patient was the first to contract the strain in the neurosurgery unit. The strain was then transmitted to case 4, case 5, and case 6 through the treatment activities of the physical therapist. The two mattress strains (20W01 and 20W02) suggested that the mattress, which was ignored for clinical sterilization for a long time, played a crucial transmission role. The hospital immediately replaced all the beds and mattresses in neurosurgery unit and ICU after the analysis. All the physician were required to disinfect their hands and instruments after each physical therapy before the subsequent treatment. The hospital also implemented strict isolation and disinfection measures for the infected, and no new cases were reported.

Briefly, this event was a nosocomial infection of ST11-K64 XDRKp, which eventually caused one death and five severe cases. The bacterium identified in this study has a wide range of antibiotic resistance. Although PFGE is the gold standard for molecular identification of epidemics, it cannot reveal the evolutionary relationship and transmission routes (Wang et al., 2020). Herein, the genome-wide information analysis technology was used to determine the source of the outbreak and reconstruct the likely transmission route of the outbreak, thus providing evidence for the prevention and control measures in hospital infection departments. The WGS result also revealed the virulence genes and drug resistance genes of the strain, explaining the poor clinical antibiotic treatment and severe symptoms caused by the infections. Our results also highlight the risk of nosocomial infections caused by ST11-K64 XDRKp. The closely monitoring and strict disinfections should be carried out to prevent the recurrence of this kind of epidemic.

## Data Availability Statement

The datasets presented in this study can be found in online repositories. The names of the repository/repositories and accession number(s) can be found at NCBI BioProject, PRJNA715018.

## Ethics Statement

Written informed consent was obtained from the individual(s), and minor(s)’ legal guardian/next of kin, for the publication of any potentially identifiable images or data included in this article.

## Author Contributions

BL designed the study, conducted bioinformatics analysis, and revised the manuscript. ZH was responsible for the coordination of laboratory resources and participated in the final revision of the manuscript. LX and LS conducted experiments, performed data analysis, and wrote the original manuscript. HT, WZ, SL, YZ, and LL performed the experiments and data analysis. All authors contributed to the article and approved the submitted version.

## Conflict of Interest

The authors declare that the research was conducted in the absence of any commercial or financial relationships that could be construed as a potential conflict of interest.

## Publisher’s Note

All claims expressed in this article are solely those of the authors and do not necessarily represent those of their affiliated organizations, or those of the publisher, the editors and the reviewers. Any product that may be evaluated in this article, or claim that may be made by its manufacturer, is not guaranteed or endorsed by the publisher.

## Footnotes

^1^ http://rast.nmpdr.org/

^2^ http://www.mgc.ac.cn/VFs/

^3^ https://card.mcmaster.ca/home

^4^ http://bigsdb.pasteur.fr/klebsiella/klebsiella.html

## Supplementary Material

The Supplementary Material for this article can be found at: https://www.frontiersin.org/articles/10.3389/fmicb.2021.709356/full#supplementary-material
